# Comorbidity Burden in Chronic Thromboembolic Pulmonary Hypertension: Implications and Outcome

**DOI:** 10.3390/medicina61050827

**Published:** 2025-04-30

**Authors:** Burcak Kilickiran Avci, Ibrahim Basarici, Mehmet Akbulut, Halil Atas, Yalin Tolga Yaylali, Umit Yasar Sinan, Ersan Atahan, Murat Meric, Baris Kaya, Kardelen Ohtaroglu Tokdil, Ozden Calay, Hasan Tokdil, Bulent Mutlu, Mehmet Serdar Kucukoglu, Zeki Ongen

**Affiliations:** 1 Department of Cardiology, Cerrahpasa Faculty of Medicine, Istanbul University-Cerrahpasa, Istanbul 34098, Turkey; kardelenohtaroglu@gmail.com (K.O.T.); hasantokdil@gmail.com (H.T.); z_ongen@yahoo.com.tr (Z.O.); 2 Department of Cardiology, Faculty of Medicine, Akdeniz University, Antalya 07070, Turkey; ibasarici@gmail.com; 3 Department of Cardiology, Faculty of Medicine, Fırat University, Elazig 23119, Turkey; drakbulut@hotmail.com; 4 Department of Cardiology, Pendik Training and Research Hospital, Marmara University, Istanbul 34899, Turkey; dratashalil@gmail.com (H.A.); bulent.mutlu@marmara.edu.tr (B.M.); 5 Department of Cardiology, Faculty of Medicine, Pamukkale University, Denizli 20160, Turkey; yaylalimd@gmail.com; 6 Department of Cardiology, Istanbul University-Cerrahpaşa Cardiology Institute, Istanbul 34098, Turkey; drumityasar@hotmail.com (U.Y.S.); kucukoglu3@yahoo.com (M.S.K.); 7 Department of Pulmonary Medicine, Cerrahpasa Faculty of Medicine, Istanbul University-Cerrahpasa, Istanbul 34098, Turkey; ersanatahan@gmail.com; 8 Department of Cardiology, Faculty of Medicine, Ondokuz Mayıs University, Samsun 55270, Turkey; drmeric@hotmail.com; 9 Department of Cardiology, Hacettepe University, Ankara 06230, Turkey; doctorkaya@yahoo.com; 10 Department of Biostatistics and Informatics Medicine, Cerrahpasa Faculty of Medicine, Istanbul University-Cerrahpasa, Istanbul 34098, Turkey; ozdencalay@gmail.com

**Keywords:** chronic thromboembolic pulmonary hypertension, comorbidity, comorbidity burden, pulmonary endarterectomy, survival

## Abstract

*Background and Objectives*: Comorbidities, the coexistence of additional conditions with a primary disease, are increasingly prevalent, complicating disease management and clinical outcomes. While CTEPH is a well-studied condition in terms of risk factors and outcomes, the specific impact of comorbidity burden on clinical presentation, treatment decisions, and survival remains insufficiently explored. This study aims to assess the prevalence and burden of comorbidities in CTEPH and to examine their associations with initial clinical characteristics, treatment allocation, and survival, stratified by pulmonary endarterectomy (PEA) status. *Materials and Methods*: We included 187 CTEPH patients from eight tertiary PH centers (2009–2020). Cardiovascular and non-cardiovascular comorbidities were identified and categorized as 0, 1–2, or ≥3. Their impact on baseline six-minute walk distance (6MWD), hemodynamic parameters, operability decision, and survival was assessed. *Results*: Comorbidities were prevalent (90%), with 49% of patients having three or more. Hypertension, diabetes, coronary artery disease, and chronic kidney disease (CKD) were associated with lower 6MWD. Hypertension, atrial fibrillation, left heart failure, and CKD were linked to elevated right atrial and pulmonary arterial wedge pressures. Comorbidities rendered 39% of anatomically operable patients ineligible for surgery. No single comorbidity predicted survival. Among PEA patients, those with ≥3 cardiovascular comorbidities had worse survival (*p* = 0.010). In contrast, the comorbidity burden did not impact survival in non-PEA patients. PEA surgery (HR 0.342, 95% CI 0.130–0.899, *p* = 0.030) and baseline 6MWD (HR 0.997, 95% CI 0.994–1.000, *p* = 0.036) were identified as independent predictors of mortality. *Conclusions*: A high comorbidity burden is common in CTEPH and influences functional status, hemodynamics, and operability decisions. It may worsen long-term outcomes after PEA but appears to be less prognostic in non-operated patients, where disease severity seems to be the primary determinant of outcomes. These findings underscore the importance of careful operability assessment and proactive comorbidity management.

## 1. Introduction

Comorbidity refers to the coexistence of one or more additional conditions with a primary disease of interest. It is increasingly common, particularly in the elderly population, and it complicates both the assessment and management of chronic diseases, often contributing to poorer clinical outcomes. In pulmonary hypertension (PH), comorbidities have been primarily discussed in the context of pulmonary arterial hypertension (PAH) [1,2,3], which is reflected in the most recent European Society of Cardiology (ESC) guideline emphasizing the importance of comorbidities in both the diagnosis and treatment decisions for PAH [4]. Similarly, the 7th World Symposium on Pulmonary Hypertension highlighted the ongoing challenges in classifying PH patients with comorbid conditions. Nonetheless, the specific treatment recommendations remained uniform for all PAH patients, regardless of comorbidity status [5].

Chronic thromboembolic pulmonary hypertension (CTEPH) represents a distinct and potentially curable subtype of PH. Numerous registries and cohort studies have described the demographic characteristics, treatment strategies, and outcomes of CTEPH patients [6,7,8,9,10,11,12,13,14,15,16]. While existing evidence indicates that individual comorbidities are common, there are limited data on the cumulative comorbidity burden and its impact on clinical presentation and outcomes, particularly when considering the two distinct clinical expressions of CTEPH: patients who undergo pulmonary endarterectomy (PEA) and those who do not.

Therefore, the present study aims to investigate the prevalence and clinical implications of comorbidities in a multicenter, real-world cohort of patients with CTEPH. Specifically, it examines the association between comorbidity burden—both individual and cumulative—and functional status, hemodynamic parameters, treatment allocation (particularly eligibility for PEA), and long-term survival, with the analyses stratified by PEA status.

## 2. Materials and Methods

This was a retrospective, multicenter, observational cohort study conducted across eight tertiary PH referral centers in Turkey. Patients diagnosed with CTEPH between January 2009 and December 2020 were included. All participating centers had established experience in the diagnosis and management of PH and followed current international guidelines during the study period [17,18].

Eligible patients were adults (≥18 years) with a confirmed diagnosis of CTEPH which required meeting three criteria: (1) evidence of pulmonary embolism (PE) with at least one mismatched segmental perfusion defect on ventilation/perfusion (V/Q) scintigraphy, confirmed by CT pulmonary angiography or invasive pulmonary angiography; (2) mean pulmonary artery pressure (mPAP) ≥ 25 mmHg on right heart catheterization (RHC); (3) these tests were performed after at least 3 months of effective anticoagulation therapy without PH-specific therapy.

The variables of interest extracted from the patient files and included initial patient characteristics, comorbidities, treatment modalities, assessments of initial and first-year World Health Organization (WHO) functional class (FC), data derived from RHC, results of pulmonary function testing, initial, and first-year six-minute walk tests (6MWT), and blood chemistry profiles including NT-proBNP or BNP. The study was conducted in accordance with the Declaration of Helsinki and approved by the Human Research Ethics Committee of the Cerrahpasa Faculty of Medicine (approval number: 83045809-604.01.02). Due to the retrospective nature of the study, informed consent was waived.

Among the eight centers, only one had the infrastructure and surgical team necessary to perform pulmonary endarterectomy (PEA). Patients from the remaining centers were referred to PEA-performing surgeons in Turkey. Initial treatment plans, including medical therapy and referral for surgery, were determined by the local multidisciplinary PH teams in each respective center. Final decisions on operability were made by the surgical teams at the referral centers.

Patients were grouped according to their PEA status as follows: the PEA (+) group included those who underwent PEA, while the PEA (−) group comprised patients who did not undergo this procedure, regardless of operability. Specific reasons for patients with anatomical suitability for PEA but not undergoing the procedure were questioned, including patient refusal, presence of comorbidities, or other factors. Patients who underwent balloon pulmonary angioplasty (BPA) procedure and were using PH-specific medications (endothelin receptor antagonists [ERAs], riociguat, phosphodiesterase-5 inhibitors [PDE5-I], inhaled iloprost, IV/SC prostacyclin) for PH were also recorded.

We categorized any previous conditions related to CTEPH as ‘associated medical conditions’ and did not consider them as comorbidities, except for acute cancer diagnosed at the time of CTEPH diagnosis. The associated medical conditions encompassed a variety of health conditions, including a history of acute PE, recurrent PE, deep vein thrombosis, previous malignancy, presence of a pacemaker, intracardiac mass, right heart infective endocarditis, splenectomy, thrombophilic disorder, myeloproliferative disorder, antiphospholipid syndrome, inflammatory bowel disease, and the presence of permanent catheters.

Comorbidities were categorized into all, cardiovascular, and non-cardiovascular groups, with each group further divided into subgroups for ‘comorbidity burden’ as those with 0, 1–2, and ≥3 comorbidities. Eight cardiovascular comorbidities were evaluated, including systemic hypertension, diabetes mellitus, active smoking, obesity (defined as a BMI equal to or greater than 30 kg/m^2^), atrial fibrillation (AF), coronary artery disease (CAD) (characterized by either greater than 50% stenosis in at least one major coronary artery or a history of percutaneous coronary intervention or coronary artery bypass grafting), heart failure (HF) (defined as a left ventricular (LV) ejection fraction less than 50% and/or PAWP or LVEDP equal or higher than 16 mmHg on RHC), and the history of stroke. Additionally, seven non-cardiovascular comorbidities were assessed, including a history of thyroid replacement therapy, anemia (defined as hemoglobin levels less than 13.5 mg/dL in men and 12.0 mg/dL in women), chronic kidney disease (CKD) (defined as an eGFR < 60 mL/dk/1.73 m^2^), lung disease (chronic obstructive pulmonary disease (COPD), asthma, severe emphysema or interstitial lung disease), connective tissue disease, and active cancer.

Individual comorbidities and comorbidity burden were evaluated across all patient groups, including those who had PEA and those who did not. Additionally, we assessed the association of initial hemodynamic parameters and 6 min walk distance (6MWD) with individual comorbidities and comorbidity burden as an answer to the question of any impact of comorbidities on clinical presentation.

### 2.1. Outcomes

All-cause mortality was the endpoint. Survival was estimated from the diagnostic RHC procedure to the date of mortality or last follow-up. The last follow-up is defined as a documented patient visit within six months prior to 1 December 2021. If a follow-up visit was not documented, mortality information was obtained through phone or electronic records. The effect of cardiovascular and non-cardiovascular individual comorbidities and comorbidity burden on survival was evaluated in both PEA (+) and PEA (−) patients. Additional outcome measures included assessing the association between comorbidity burden and improvements in WHO Functional Class (WHO-FC) and changes in 6 min walk distance (6MWD) during the initial year (∆6MWD) compared to baseline values. Improvement in WHO-FC is defined as any class improvement from the baseline value.

### 2.2. Statistics

Statistical analysis was performed using IBM SPSS version 29.0. A *p*-value less than 0.05 was considered statistically significant. Baseline characteristics were presented as counts and percentages for discrete variables and as mean ± SD for continuous data. Categorical variables were compared using chi-squared or Fisher’s exact tests, as appropriate. Continuous data were analyzed using Student’s *t*-test for normally distributed data and the Mann–Whitney U test for non-normally distributed data. Multiple group comparisons were conducted using one-way ANOVA followed by Tukey’s multiple comparison test. Survival analysis was performed using Kaplan–Meier estimates and Cox proportional hazards regression, presenting results as hazard ratios (HR) with 95% confidence intervals.

## 3. Results

We enrolled a total of 187 patients, with the majority being female (63.1%) and a mean age of 60 years. Most patients presented with WHO-FC III symptoms at baseline (71.7%). The mPA P was 41.4 ± 11.0 mmHg, and the PVR was 7.7 ± 4.5 WU. PAWP was >15 mmHg in 26 (13.9%) patients. Further details on patient characteristics and associated medical conditions can be found in Table 1.

### 3.1. Treatment Strategies

Among the 187 CTEPH patients, 59 had surgically inaccessible disease, while the remaining 128 were considered anatomically operable. Of these, 64 underwent PEA surgery [PEA (+)], while the remaining 64 patients did not. The primary reasons for not undergoing surgery included patient refusal (33 patients), morbid obesity (9 patients), COPD (5 patients), severe CAD (5 patients), HF (3 patients), CKD (3 patients), and unknown reasons (6 patients). Thus, the PEA (−) group comprised 123 patients, including those with surgically inaccessible disease and others with accessible disease who did not undergo surgery. BPA was performed in 15 patients, with 6 in the PEA (+) group and 9 in the PEA (−) group.

The PEA (+) group, as detailed in Table 1, exhibited a younger age and a higher prevalence of previous acute PE, DVT, and antiphospholipid syndrome compared to the PEA (−) group. Despite a more compromised hemodynamic profile with higher sPAP, mPAP, and PVR, they demonstrated better performance on the initial 6MWD test compared to those in the PEA (−) group.

PH-specific therapy was administered to 73.8% of the overall cohort during follow-up. Use of such treatment was significantly higher in the PEA (−) group compared to the PEA (+) group (82.1% vs. 48.4%, *p* < 0.001). Riociguat was the most frequently prescribed agent (65.2%), followed by ERAs (15.6%). The use of PDE5-I, inhaled iloprost, and IV/SC prostacyclins was relatively less frequent. Detailed data on PH-specific therapies and their distribution across groups are presented in Table 1.

Anticoagulation therapy was administered to the vast majority of patients (96.3%), and this rate was similar between the PEA (+) and PEA (−) groups (95.3% vs. 96.7%, *p* = 0.578). Only one patient in the PEA (−) group was documented to be receiving additional long-term antiplatelet therapy (acetylsalicylic acid [ASA]). No further data were available on temporary antiplatelet use, such as following myocardial infarction or stent placement.

### 3.2. Comorbidities

**Figure 1:**
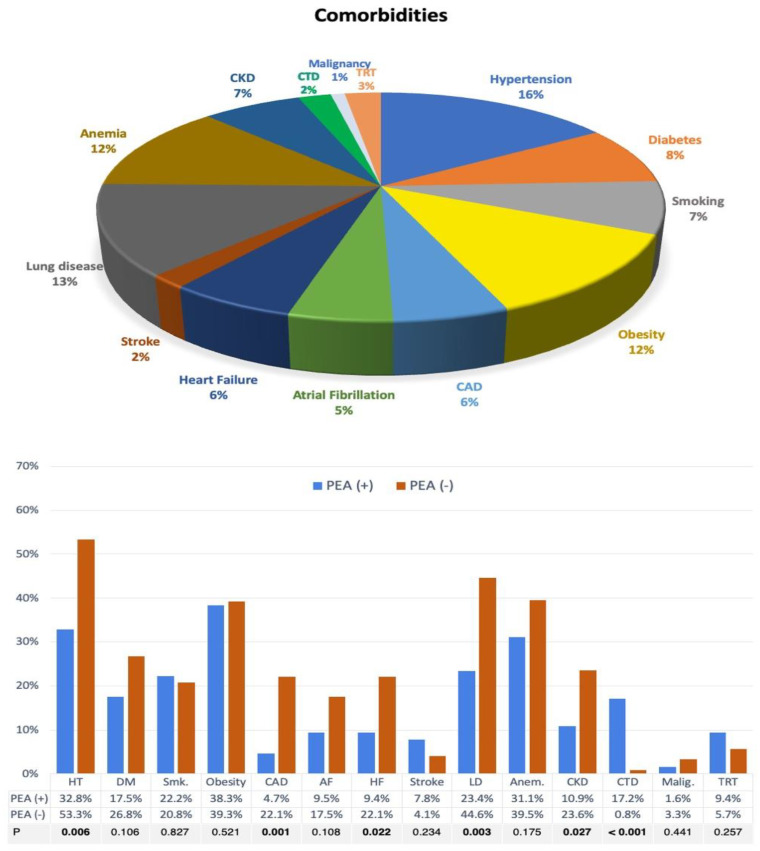
Prevalance of individual comorbidities in entire cohort and according to PEA status. AF: atrial fibrillation, Anem.: anemia, CAD: coronary artery disease, CKD: chronic kidney disease, CTD: connective tissue disease, DM: diabetes mellitus, HT: hypertension, LD: lung disease, Malig.: active malignancy, Smk.: smoking, TRT: thyroid replacement therapy.

The prevalence of comorbidities among the study participants was notably high, with nine in ten patients having one or more comorbidities and 72% being multimorbid. (Figure 1 and Suppl. Table S1) The most common comorbidities included systemic hypertension (46%), lung disease (36.9%), anemia (35.3%), and obesity (34.8%). Cardiovascular comorbidities were more prevalent than non-cardiovascular comorbidities (78% vs. 67%, respectively).

Comorbidities were further analyzed in relation to PEA (+) or PEA (−) status, with hypertension, CAD, HF, lung disease, and CKD being significantly more prevalent in the PEA (−) group compared to the PEA (+) group. (Figure 1 and presented in Suppl. Table S1). Conversely, connective tissue disease was notably more common in the PEA (+) group. Additionally, the number of comorbidities was higher among patients in the PEA (−) group compared to the PEA (+) group.

### 3.3. The Relationship of Comorbidities with Baseline Hemodynamics and 6MWD

The presence of individual comorbidities such as hypertension, diabetes, CAD, and CKD was associated with worse initial 6MWD (Suppl. Table S2). In terms of hemodynamics, hypertension, AF, HF, and CKD were associated with increased RAP, while hypertension, HF, CKD, and anemia were associated with increased PAWP (Suppl. Table S3). PVR was lower in the presence of obesity, HF, or active cancer.

According to comorbidity burden, patients with three or more comorbidities were found to be older, had worse 6MWD, and exhibited increased RAP and PAWP compared to those with no or one to two comorbidities (Table 2). However, there were no significant differences observed in sPAP, mPAP, PVR and CI among the groups. Although undergoing PEA was more common in patients with no comorbidity, PH-specific treatment and combination treatment usage did not differ between these groups.

### 3.4. Outcome

#### 3.4.1. Survival

The median follow-up duration was 41.2 months. During this period, 44 patients died, with 3 (4.7%) of these deaths occurring perioperatively. During follow-up, 7 (11.5%) in the PEA (+) group and 34 (27.6%) in the PEA (−) group were deceased (*p* = 0.010). The Kaplan–Meier analysis shows better survival in operated patients (*p* < 0.001). Survival estimates at one and three years were higher in the PEA (+) group than in the PEA (−) group (98.4% and 88.2% versus 87.8% and 76.7%, respectively). In the Cox-regression analysis, PEA status (HR 0.342, 95% CI 0.130–0.899, *p* = 0.030) and initial 6MWD (HR 0.997, 95% CI 0.994–1.000, *p* = 0.036) emerged as independent determinants of mortality among considered parameters, including age, sPAP, PEA status, and initial 6MWD.

**Figure 2:**
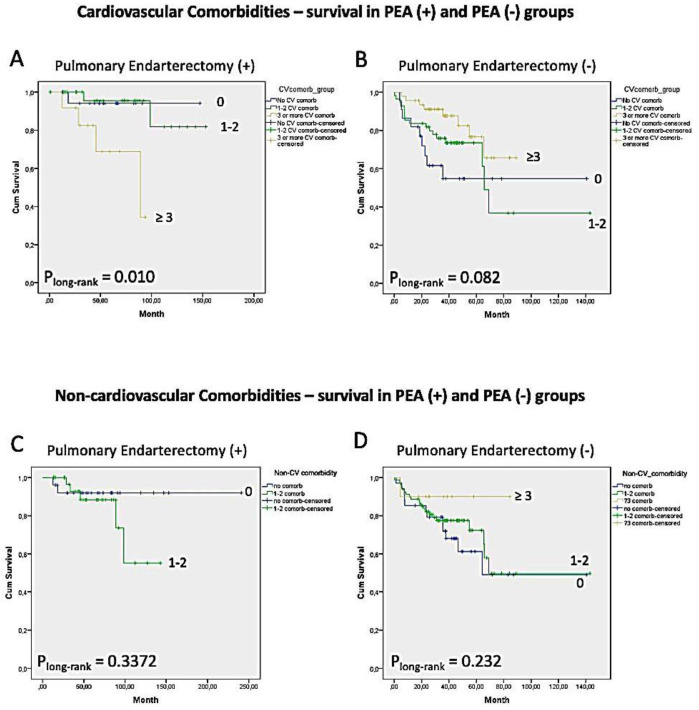
Kaplan–Meier survival estimates based on cardiovascular (**A**,**B**) and non-cardiovascular (**C**,**D**) Comorbidity Burden in PEA (+) and PEA (−) Patients.

Regarding PEA and cardiovascular comorbidity burden status, in the PEA (+) group, patients with ≥3 cardiovascular comorbidities had worse survival compared to those with 1–2 comorbidities or none (*p* = 0.010) (Figure 2). No significant survival difference was observed in the PEA (−) group. Similarly, the non-cardiovascular comorbidity burden was not associated with decreased survival in either PEA (+) or PEA (−) patients.

#### 3.4.2. Survivors vs. Non-Survivors

Table 3 displays the baseline characteristics of survivors and non-survivors in the entire cohort. Survivors were younger and exhibited better initial (291 vs. 209 m, *p* = 0.001) and first-year (359 vs. 236 m, *p* < 0.001) 6MWD values. They also more commonly underwent PEA surgery (38% vs. 17%, *p* = 0.013). Neither the individual comorbidities investigated nor the burden of comorbidities differed between survivors and non-survivors (Table 3).

We further analyzed the differences in baseline characteristics of survivors and non-survivors concerning PEA status (Supplementary Tables S4 and S5). In the PEA (+) group, survivors were younger and exhibited higher initial (325 m vs. 179 m) and first-year (426 m vs. 207 m) 6MWD measurements, along with a lower number of comorbidities (mean count 1.9 vs. 3.7, *p* = 0.002). They also had a decreased prevalence of CAD (1.9% vs. 28.6%) and AF (5.7% vs. 42.9%). No significant differences were observed in the long-term use of PH-specific medications.

In PEA (−) CTEPH group, survivors exhibited a better hemodynamic profile, evidenced by lower sPAP: (59 mmHg vs. 70 mmHg, *p* = 0.004), mPAP (37 mmHg vs. 42 mmHg, *p* = 0.014) and PVR (6.5 WU vs. 8.4 WU, *p* = 0.041) compared to non-survivors. Initial (269 m vs. 217 m, *p* = 0.056) and first-year (315 m vs. 246 m, *p* = 0.21) 6MWD were also better among survivors compared to non-survivors. Lung diseases were significantly more prevalent among survivors (52% vs. 24%, *p* = 0.007), with a trend toward higher incidence of AF (21.6% vs. 6.3%, *p* = 0.059). Interestingly, survivors in this group exhibited a higher burden of comorbidities (mean count 3.3 vs. 2.4, *p* = 0.007) compared to non-survivors. Although not significant, the mortality ratio was lower in patients with ≥3 comorbidities compared to 1–2 and none groups (19.1% vs. 38.8% vs. 33.3%, *p* = 0.061) (Table 2). No significant differences were noted in the utilization of PH-specific medications between survivors and non-survivors (83% vs. 79%) within the PEA (−) group.

### 3.5. 6MWD and WHO-FC in First Year of Follow-Up

The results of the 6MWT1styear were available in 72% of patients. Among them, 6MWT1styear performance was worse in patients with ≥3 comorbidities in the entire cohort and in the PEA (+) group (Table 2). However, according to comorbidity status, we did not find any significant difference in Δ6MWD or FC improvement relative to the initial value at the first year in the entire cohort or according to PEA status.

## 4. Discussion

This multicenter, real-world study yielded several findings regarding the prevalence and impact of comorbidities in patients with CTEPH. First, comorbidities were highly prevalent, with nearly half of the cohort presenting with three or more coexisting conditions. Second, the pulmonary endarterectomy (PEA) rate was relatively low, with only one-third of patients undergoing surgery—substantially lower than reported in international registries and some single-center reports, particularly those based in high-volume surgical centers [6,8,12,14,16]. In many cases, patient refusal and comorbidities were the primary reasons for not proceeding with surgery despite technical operability. Third, both individual comorbidities and cumulative comorbidity burden were associated with reduced exercise capacity and more complex hemodynamic profiles, including elevated RAP and PAWP. Fourth, in line with previous studies, PEA was associated with improved survival, while a higher comorbidity burden appeared to negatively impact long-term outcomes in operated patients.

We compared our findings with the previous registries reporting individual comorbidity data [6,7,8,9,10,11,12,13,16] (Table 4). The age distribution in our cohort was similar to most previous studies [6,7,8,10,13,14,16]. However, the prevalence of female patients was higher than in some larger registries [6,9,10,12,14,16] but similar to Japanese, Korean, and Mexican cohorts [7,8,13]. The most common comorbidities in our cohort were systemic hypertension (46%), followed by lung disease (36.9%), anemia (35.3%), and obesity (34.8%) in our cohort. Overall, the prevalence of individual comorbidities in our cohort was higher than in most of the registries, except for history of hypertension [10], any cancer [6,12,16], obesity [6,7], CKD [16], and thyroid disorders [10,16], which were more common in the referenced registries.

Nearly half of the CTEPH patients in our study had three or more comorbidities. Both individual comorbidities and the overall comorbidity burden seemed to affect clinical presentation, leading to reduced 6MWD and elevated RAP and PAWP. This was especially true for cardiovascular comorbidities but also for some non-cardiovascular comorbidities, such as anemia and active cancer. Elevated PAWP indicates a more complex or mixed PH profile in some CTEPH patients, potentially reflecting group 2 (left-heart associated) PH features. In our cohort, 13.9% of patients had increased PAWP. Gerges C et al. reported that increased left ventricle filling pressure (>15 mmHg) was 10.6% of CTEPH patients in their cohort and was associated with increased long-term mortality [16]. We could not find a direct association of PAWP with long-term survival, possibly due to the smaller size of our cohort. They also highlighted multiple cardiovascular comorbidities, including systemic hypertension, diabetes, AF, calcific aortic valve stenosis, and mitral regurgitation in relation to increased filling pressures. In our cohort, the presence of hypertension, HF, CKD, and anemia were associated with higher PAWP.

Comorbidities also seem to affect therapeutic strategies. In our cohort, half of the patients with surgically accessible disease did not undergo the procedure. We found that a high comorbidity burden was associated with a low prevalence of surgery. Among those who did not undergo surgery, 39% cited severe comorbidities as the primary reason for not proceeding with PEA. Lung diseases, morbid obesity, CAD, HF, and CKD were the main comorbidities for technically operable patients who did not undergo surgery. In comparison, the US-CTEPH Registry reported comorbidities as the reason for non-surgical management in 19% of technically operable patients, citing COPD, left ventricular dysfunction, and hematologic malignancies [6]. Similarly, the International Registry reported that 19.6% of operable patients were not referred for surgery due to comorbidities [14]. These findings highlight the significant impact of comorbidities on the decision-making process for surgical intervention in CTEPH patients.

The lower PEA rate in our cohort and the higher rate of comorbidity-related surgical ineligibility compared to other studies may be partly explained by the greater comorbidity burden in our population. Additionally, the absence of dedicated PEA programs in most participating centers and the limited number of surgical centers dedicated to PEA in our country may also contribute to the lower surgical rate.

These observations may also offer insight into why some studies report lower PEA rates. In healthcare systems where access to PEA is limited and nationwide referral is not mandatory, patients may be deprived of potentially curative surgical treatment. Our findings highlight the need to strengthen referral pathways and ensure equitable access to surgery.

Our survival analysis aligns with existing literature, showing better long-term outcomes in patients who underwent PEA, with the procedure serving as an independent predictor of survival [6,8,15]. We did not find any impact of individual cardiovascular and non-cardiovascular comorbidities on survival in the whole cohort; however, our study cohort was too small to draw definitive conclusions. Notably, PEA (+) patients with a higher cardiovascular comorbidity burden (three or more comorbidities) exhibited poorer survival outcomes. This underscores the importance of considering the comorbidity burden in post-PEA care and focusing treatment on managing these comorbidities. In contrast, patients who did not undergo PEA demonstrated worse survival compared to PEA (+) patients despite having a better initial hemodynamic profile. These patients were older, had worse 6MWT performance, and exhibited a greater comorbidity burden. Interestingly, in the non-operated group, we did not observe a clear association between specific comorbidities or overall comorbidity burden and survival; rather, deceased patients tended to have more severe pulmonary hemodynamics and worse 6MWD but fewer comorbidities.

These findings suggest that, after PEA surgery, comorbidity burden may become the major determinant of long-term prognosis. Conversely, for patients who did not undergo PEA surgery, it can be speculated that the natural course of CTEPH remains the main contributor to survival, with individual comorbidities or overall comorbidity burden having a lesser impact. This distinction underscores the need for comprehensive and individualized operability assessments, as PEA remains the most effective treatment for improving survival in CTEPH.

### Limitations

This study has several limitations. Its retrospective design and the broad inclusion period (2009–2020) may have introduced heterogeneity in diagnostic and treatment approaches. However, all patients met the standardized diagnostic criteria for CTEPH, including documented mismatched perfusion defects, confirmatory findings on CT or invasive pulmonary angiography, and hemodynamic confirmation of PH via RHC after an adequate course of anticoagulation, in line with international guideline recommendations [17,18].

BPA use was limited during the study period due to a lack of reimbursement and operator experience. Operability assessments were not standardized across centers, and access to PEA on-site was limited, potentially influencing treatment allocation compared to high-volume surgical registries. Nonetheless, these variations reflect real-world practice and offer insights into everyday management challenges.

We assessed comorbidity burden based on the number of comorbidities, which is not the most precise method for evaluating individual comorbidity impact. Unfortunately, no perfect method exists. While indices such as the Charlson Comorbidity Index or Elixhauser Comorbidity Scores offer more detailed assessments, our retrospective design limited their use [19,20]. Additionally, missing pulmonary function data prevented further analysis of their effect on PH and outcomes.

## 5. Conclusions

Comorbidities and high comorbidity burden are common in CTEPH and contribute to a more complex clinical and hemodynamic profile, often incorporating features of group 2 PH features. Comorbidities impact the baseline functional and hemodynamic status and negatively influence decisions regarding surgical referral and operability. Rather than individual comorbidities, the overall comorbidity burden may have a greater impact on post-PEA survival. Conversely, for patients who do not undergo PEA surgery, CTEPH itself appears to be the primary driver of prognosis. These findings reinforce the importance of comprehensive operability evaluation and suggest that optimizing comorbidity management may further improve outcomes, particularly in patients undergoing surgical intervention.

## Figures and Tables

**Table 1:** Baseline clinical and hemodynamic characteristics based on PEA status.

	Total N = 187	PEA (+) N = 64	PEA (−) N = 123	*p* Value
***Demographics***				
Age	60.2 ± 14.6	51.6 ± 14.1	64.7 ± 12.8	<0.001
Female	118 (63.1)	38 (59.4)	80 (65.0)	0.273
***Associated medical conditions***				
Previous acute PE history	129 (69.0)	57 (89.1)	72 (59.5)	<0.001
Deep vein thrombosis history	56 (30)	27 (42.2)	29 (23.6)	0.008
Malignancy history	12 (6.5)	2 (3.2)	10 (8.3)	0.156
Active malignancy	5 (2.7)	1 (1.6)	4 (3.3)	0.434
Pacemaker	3 (1.6)	0 (0)	3 (2.5)	0.275
Intracardiac mass	3 (1.6)	1 (1.6)	2 (1.7)	0.722
Right infective endocarditis	1	0 (0.5)	1 (0.8)	0.648
Splenectomy	2 (1.1)	0 (0)	2 (1.7)	0.426
Thrombophilic disorder	21 (12.1)	11 (18)	10 (8.9)	0.080
Antiphospholipid syndrome	8 (4.6)	8 (13.1)	0 (0)	<0.001
Inflammatory bowel disease	4 (2.2)	1 (1.6)	3 (2.5)	0.567
***Clinical Findings***				
Body mass index, kg/m^2^	29.4 ± 6.1	28.7 ± 4.9	29.9 ± 6.7	0.225
WHO-FC I	1 (0.5)	0 (0)	1 (0.8)	0.493
II	34 (18.2)	14 (21.9)	20 (16.3)	
III	134 (71.7)	46 (71.9)	88 (71.5)	
IV	18 (9.6)	4 (6.3)	14 (11.4)	
6MWD, m [patient number]	278 ± 145 [164]	309 ± 143 [58]	261 ± 144 [106]	0.044
***Hemodynamics***				
Mean RA pressure, mmHg	10.9 ± 4.8	11.2 ± 5.3	10.7 ± 4.5	0.569
PA systolic pressure, mmHg	67.2 ± 20.5	76.8 ± 20.7	62.2 ± 18.6	<0.001
PA mean pressure, mmHg	41.4 ± 11.0	46.6 ± 11.7	38.6 ± 9.6	<0.001
PA wedge pressure, mmHg	11.6 ± 4.8	11.2 ± 11.0	11.8 ± 11.0	0.397
PVR, Wood units	7.7 ± 4.5	9.1 ± 4.3	7.0 ± 4.4	0.001
Cardiac index, L/min/m^2^	2.4 ± 0.8	2.3 ± 0.7	2.5 ± 0.8	0.380
***Medical therapy***				
PH-specific therapy at any time	138 (73.8)	37 (57.8)	101 (82.1)	<0.001
Riociguat	122 (65.2)	31 (48.4)	92 (75.4)	<0.001
Phosphodiesterase 5 inhibitors	10 (5.3)	3 (4.7)	7 (5.7)	0.529
ERAs	29 (15.6)	8 (12.5)	21 (17.2)	0.412
Iloprost inhaler	8 (4.3)	7 (10.9)	1 (0.8)	0.003
IV/Sc prostacycline	5 (2.7)	2 (3.1)	3 (2.5)	0.562
Anticoagulation	180 (96.3)	61 (95.3)	119 (96.7)	0.578

6MWD: six-minute walk distance, APAS: antiphospholipid antibody syndrome, BPA: balloon pulmonary angioplasty, PA: pulmonary artery, PE: pulmonary embolism, PEA: pulmonary endarterectomy, PH: pulmonary hypertension, PVR: pulmonary vascular resistance, WHO-FC: World Heart Organization-functional class.

**Table 2:** Patient clinical and hemodynamic characteristics and outcomes based on comorbidity burden.

	N/N/N	No Comorbidity ^a^	1–2 Comorbidity ^b^	3 or More Comorbidity ^c^	*p*
Age, years	16/79/92	46.2 ± 17.4	56.5 ± 14.5	65.8 ± 11.3	<0.001 *
Age groups	16/79/92				<0.001
18–45 years		10 (62.5)	20 (25.3)	6 (6.5)	
46–65 years		3 (18.8)	34 (43.0)	37 (40.2)	
≥66 years		3 (18.8)	25 (31.6)	49 (53.3)	
Female, n (%)	16/79/92	9 (56.3)	50 (63.3)	59 (64.1)	0.631
WHO-FC III or IV	16/79/92	11 (68.8)	66 (83.5)	74 (80.5)	0.638
6MWD, m	16/66/85	359.4 ± 150.2	301.3 ± 148.4	234.8 ± 137.6	<0.001 ^#^
ProBNP	11/47/68	1591 ± 1554	785 ± 1123	2087 ± 4234	0.109
BNP	0/11/16	-	378 ± 562	571 ± 1240	0.634
***Treatment***					
Pulmonary endarterectomy	16/79/92	10 (62.5)	30 (38.0)	24 (26.1)	0.007
Balloon pulmonary angioplasty	16/79/92	1 (6.3)	3 (3.8)	5 (5.4)	0.849
PH-specific treatment	16/78/92	12 (75.0)	57 (72.2)	69 (75.0)	0.768
Riociguat		11 (68.8)	51 (65.4)	61 (66.3)	0.965
PDE5-I		0 (0)	4 (5.1)	6 (6.5)	0.368
ERAs		5 (31.2)	7 (9.0)	17 (18.5)	0.05
Iloprost inh		0 (0)	3 (3.8)	5 (5.4)	0.425
IV/Sc prostacycline		0 (0)	1 (1.3)	4 (4.3)	0.298
Combination treatment	16/78/92	4 (25.0)	10 (12.7)	19 (20.7)	0.561
***Hemodynamics***					
Mean RA pressure, mmHg	16/78/91	8.2 ± 4.2	9.8 ± 4.3	12.3 ± 4.8	<0.001 ^†^
PA systolic pressure, mmHg	16/79/92	77.6 ± 17.7	65.2± 20.0	67.0 ± 21.1	0.086
PA mean pressure, mmHg	16/79/92	46.1 ± 10.9	40.2 ± 10.3	41.5 ± 11.5	0.149
PA wedge pressure, mmHg	16/75/89	9.2 ± 3.1	10.3 ± 3.4	13.2 ± 5.6	<0.001 ^§^
PVR, WU	16/78/90	8.5 ± 2.5	8.1 ± 4.6	7.3 ± 4.6	0.372
Cardiac index, L/min/m2	15/65/87	2.5 ± 0.8	2.4 ± 0.8	2.3 ± 0.8	0.716
**OUTCOMES of all and according to PEA status**					
**Outcomes of all patients**					
First-year 6MWD, m	14/50/70	443 ± 99	361 ± 137	295 ± 140	<0.001 ^¢^
First-year 6MVD, m	14/45/68	80.0 ± 117.6	72.7 ± 135.3	51.5 ± 118.5	0.578
First-year FC improved n (%)	15/62/78	8 (53.3)	39 (62.9)	38 (48.7)	0.244
Mortality n (%)	16/78/90	3 (18.8)	19 (24.4)	19 (21.1)	0.893
**Outcomes of PEA (+) patients**					
First-year 6MWD, m	9/21/20	470 ± 76	420 ± 111	334 ± 172	0.030 ^£^
First-year 6MVD, m	10/25/18	95 ± 120	87.9 ± 104.5	94.7 ± 165.5	0.984
First-year FC improved n (%)	32/21	5 (50.0)	16 (72.7)	12 (57.1)	0.387
Mortality n (%)	10/30/24	1 (10.0)	0 (0)	6 (27.3)	0.010
**Outcomes of PEA (−) patients**					
First-year 6MWD, m	5/29/50	394 ± 125	318 ± 140	280 ± 123	0.119
First-year 6MVD, m	5/25/49	53 ± 121.6	60.5 ± 156.7	34.8 ± 91.0	0.663
First-year FC improved n (%)	5/40/57	3 (60.0)	23 (57.5)	26 (45.6)	0.472
Mortality n (%)	6/49/68	2 (33.3)	19 (38.8)	13 (19.1)	0.061

N/N/N represents the number of patients with no comorbidity/1 or 2 comorbidities/≥3 comorbidites. 6MWD: change in six-minute walk distance relative to the initial 6MWD measurement, PA: pulmonary artery, PEA: pulmonary endarterectomy, PVR: pulmonary vascular resistance, RA: right atrium, FC: world heart organization-functional class. * a vs. b *p* = 0.015, a vs. c *p* < 0.001, b vs. c *p* < 0.001, ^#^ a vs. c *p* = 0.005, b vs. c *p* = 0.016. ^†^ a vs. c *p* = 0.004, b vs. c *p* = 0.002, ^§^ a vs. c *p* = 0.005, b vs. c *p* < 0.001, ^¢^ a vs. c *p* < 0.001, b vs. c *p* = 0.031, ^£^ a vs. c *p* = 0.048.

**Table 3:** Characteristics of Survivors and Non-survivors in the Entire Cohort.

	N/N	Survivors N = 143	Non-Survivors N = 41	*p*
Age	143/41	58.5 ± 14.9	65.9 ± 12.5	0.002
Female	143/41	91 (63.6)	26 (63.4)	0.979
***Comorbidities***				
Sytemic hypertension	142/41	66 (46.5)	17 (41.5)	0.570
Diabetes mellitus	142/41	33 (23.2)	10 (24.4)	0.878
Smoking	142/41	29 (20.4)	10 (24.4)	0.585
Obesity	132/32	53 (40.2)	12 (37.5)	0.783
Coronary artery disease	143/41	24 (16.8)	6 (14.6)	0.743
Atrial fibrillation	141/39	22 (15.6)	5 (12.8)	0.667
Heart Failure	143/41	24 (16.8)	8 (19.5)	0.684
Stroke	142/41	8 (5.6)	1 (2.4)	0.686
Lung disease	143/40	56 (39.2)	11 (27.5)	0.176
Anemia	139/39	50 (36)	16 (41)	0.564
Chronic kidney disease	143/41	24 (16.8)	11 (26.8)	0.148
Connective tissue disease	143/41	12 (8.4)	0 (0)	0.071
Active cancer	143/41	4 (2.8)	1 (2.4)	1.000
Thyroid replacement therapy	143/41	11 (7.7)	2 (4.9)	0.736
***Comorbidity burden***				
Total number of comorbidities	143/41	2.8 ± 1.9	2.6 ± 1.7	0.626
≥3 comorbidites	143/41	71 (49.7)	19 (46.3)	0.709
≥3 CV comorbidities	141/41	45 (31.9)	12 (29.3)	0.748
≥3 non-CV comorbidities	143/41	9 (6.3)	1 (2.4)	0.462
***Functional capacity***				
WHO-FC I–II III–IV	143/41	31 (21.7) 112 (78.3)	4 (9.8) 37 (90.2)	0.086
Initial 6MWD, m [51/6]	133/32	290.6 ± 150.9	209.4 ± 116.0	0.001
First-year 6MWD, m [43/7]	108/26	359.2 ± 130.6	235.5 ± 150.5	<0.001
6MVD, m	105/22	69.2 ± 111.6	28.7 ± 171.6	0.269
***Hemodynamics***				
Mean RA pressure, mmHg	141/41	11.0 ± 4.9	10.2 ± 4.3	0.341
PA systolic pressure, mmHg	143/41	65.5 ± 20.7	72.1 ± 19.8	0.069
PA mean pressure, mmHg	143/41	40.5 ± 11.1	43.7 ± 10.3	0.099
PA wedge pressure, mmHg	138/39	11.4 ± 4.5	12.4 ± 5.6	0.236
PVR, Wood units	142/39	7.5 ± 4.4	8.4 ± 4.8	0.282
Cardiac index, L/min/m^2^	131/33	2.40 ± 0.76	2.35 ± 0.8	0.747
***Treatment***				
Pulmonary endarterectomy	143/41	54 (37.8)	7 (17.1)	0.013
Pulmonary vasodilator drugs	143/41	104 (72.7)	33 (80.5)	0.315

Δ6MWD: change in six-minute walk distance relative to the initial 6MWD measurement, 6MWD: six-minute walk distance, PA: pulmonary artery, PVR: pulmonary vascular resistance, RA: right atrium.

**Table 4:** Comparison of comorbidities in CTEPH registries and studies.

	International Prospect Registry 2011, 2016 [12,14]			Korean Registry Park SY, 2016 [13]	Al-Naamani, et al. A Mexican Center 2016 [7]	Miwa H, et al. A Japanese Center, 2018 [8]	PHSANZ Registry Kearney K, et al. Australia and New Zealand, 2020 [9]		US-CTEPH Registry Kerr KM, et al. 2021 [6]			Polish Registry, BNP-PL, Kopec G, et al. 2021 [10]	Swedish Registry, Kjellstrom B, et al. 2022 [11]			Gerges C, et al. An Austrian Center, 2023 [16]
	Total N = 679	PEA (+) N = 404	PEA (−) N = 275	Total N = 134	Total N = 50	Total N = 280	PEA (+) N = 146	PEA (−) N = 240	Total N = 750	PEA (+) N = 566	PEA (−) N = 184	Total N = 516	Total N = 311	PEA (+) N = 98	PEA (−) N = 213	Total N = 593
Age, mean ± SD or median [interquartile range]	63 [51–72]	60	67	58.3 ± 15.9	63 [53–75]	57 ± 12.5	55 ± 16	62 ± 16	59 [46–69]	57 [44–67]	67 [54–73]	63.8 ± 15.4	70 [62–76]	65 [52–72]	72 [65–77]	58.7 ± 15.5
Female, %	40.9	45	57	56.7	58	71	47.2	56.7	49.2	52.7	55	50.4	50	37	56	49.9
Systemic HT, %				21.6	34		26	35	36.1	35.7	37.5	58.7	41	32	45	48.7
Diabetes, %	5.2 #			6.7	14	0.7 (severe DM)	6.8	12	15.1	13.6	19.6	17	8	2	10	12
Smoking, %		34	34				43.8	33.8	40.3	38.7	45.1	4.7 (present) 27.7 (past)				
Obesity, %	17.6	17	20		40				49.2	51.1	43.5	31.6	22	19	24	18.9
Coronary artery dis., %	11.8	10	15			2.1	19.2	10.4	10.9	9.5	15.2	18.6	10	3	13	15.3
Atrial fibrillation, %					14				7.1	7.1	7	14.7	10	5	13	10.1
Heart failure, %	6.5 *	5	9	4.5 Congestive HF	12 Cardiomyopathy				3.6	2.5	7.1					
Stroke, %						6			5.5	5.1	6.5		5	7	4	
Lung disease, %	10.8 9.5 1.3 -	8	13		32	2.1			29.1	27.2	34.8					
COPD					22				15.2	12.4	23.9	9.5				
ILD					6				1.6	1.4	2.2	1.6				
Asthma				2.9	4				12.3	13.6	8.7	5.8				
Anemia, %																
Chronic kidney dis., %		0.5 dialy dep.	0.4 dialy dep.			1.8						24.6	4 (eGFR < 30)	1	6	41.8
Connective tissue dis,%				2.2					2.6	2.9	1.6					
Any cancer, %	12.7	12	16	2.2	2	5.7			9.2	7.4	14.7	1.9 (present) 7.6 (past)				13.8
Thyroid disorder and HRT %	8.4				6				11.1	11.1	11.0	12 hypothyroidism 5.6 hyperthyroidism				18.5

Continuous variables are expressed as means ± SD or as medians with [first and third quartiles], while categorical variables are expressed as percentages. PEA (+) denotes patients who underwent surgery, while PEA (−) includes both anatomically inoperable patients and those who are operable but did not undergo surgery. Dis.: disease, COPD: Chronic obstructive pulmonary disease, HRT: hormone replacement therapy, HT: hypertension, ILD: interstitial lung disease. # non-insulin dependent diabetes, * 1.9% left ventricular diastolic dysfunction, 4.6% congestive heart failure.

## Data Availability

The data presented in this study are available on request from the corresponding author.

## Supplementary Materials

The following supporting information can be downloaded at: https://www.mdpi.com/article/10.3390/medicina61050827/s1, Table S1. Co-morbidities according to PEA status; Table S2. Initial six-minute walk distance by comorbidity status; Table S3. Baseline hemodynamic parameters according to comorbidity status; Table S4. Characteristics of survivors and non-survivors in PEA (+) group; Table S5. Characteristics of survivors and non-survivors in PEA (−) group.

## References

[1] Benza R.L., Miller D.P., Gomberg-Maitland M., Frantz R.P., Foreman A.J., Coffey C.S., Frost A., Barst R.J., Badesch D.B., Elliott C.G. (2010). Predicting Survival in Pulmonary Arterial Hypertension. Circulation.

[2] Poms A.D., Turner M., Farber H.W., Meltzer L.A., McGoon M.D. (2013). Comorbid Conditions and Outcomes in Patients With Pulmonary Arterial Hypertension. Chest.

[3] Hoeper M.M., Dwivedi K., Pausch C., Lewis R.A., Olsson K.M., Huscher D., Pittrow D., Grünig E., Staehler G., Vizza C.D. (2022). Phenotyping of idiopathic pulmonary arterial hypertension: A registry analysis. Lancet Respir. Med..

[4] Humbert M., Kovacs G., Hoeper M.M., Badagliacca R., Berger R.M.F., Brida M., Carlsen J., Coats A.J.S., Escribano-Subias P., Ferrari P. (2022). 2022 ESC/ERS Guidelines for the diagnosis and treatment of pulmonary hypertension. Eur. Heart J..

[5] Kovacs G., Bartolome S., Denton C.P., Gatzoulis M.A., Gu S., Khanna D., Badesch D., Montani D. (2024). Definition, classification and diagnosis of pulmonary hypertension. Eur. Respir. J..

[6] Kerr K.M., Elliott C.G., Chin K., Benza R.L., Channick R.N., Davis R.D., He F., LaCroix A., Madani M.M., McLaughlin V.V. (2021). Results From the United States Chronic Thromboembolic Pulmonary Hypertension Registry. Chest.

[7] Al-Naamani N., Espitia H.G., Velazquez-Moreno H., Macuil-Chazaro B., Serrano-Lopez A., Vega-Barrientos R.S., Hill N.S., Preston I.R. (2016). Chronic Thromboembolic Pulmonary Hypertension: Experience from a Single Center in Mexico. Lung.

[8] Miwa H., Tanabe N., Jujo T., Kato F., Anazawa R., Yamamoto K., Naito A., Kasai H., Nishimura R., Suda R. (2018). Long-Term Outcome of Chronic Thromboembolic Pulmonary Hypertension at a Single Japanese Pulmonary Endarterectomy Center. Circ. J..

[9] Kearney K., Gold J., Corrigan C., Dhital K., Boshell D., Haydock D., McGiffin D., Wilson M., Collins N., Cordina R. (2021). Chronic thromboembolic pulmonary hypertension in Australia and New Zealand: An analysis of the PHSANZ registry. Respirology.

[10] Kopeć G., Dzikowska-Diduch O., Mroczek E., Mularek-Kubzdela T., Chrzanowski Ł., Skoczylas I., Tomaszewski M., Peregud-Pogorzelska M., Karasek D., Lewicka E. (2021). Characteristics and outcomes of patients with chronic thromboembolic pulmonary hypertension in the era of modern therapeutic approaches: Data from the Polish multicenter registry (BNP-PL). Ther. Adv. Chronic Dis..

[11] Kjellström B., Bouzina H., Björklund E., Beaudet A., Edwards S.C., Hesselstrand R., Jansson K., Nisell M., Rådegran G., Sandqvist A. (2022). Five year risk assessment and treatment patterns in patients with chronic thromboembolic pulmonary hypertension. ESC Heart Fail..

[12] Pepke-Zaba J., Delcroix M., Lang I., Mayer E., Jansa P., Ambroz D., Treacy C., D’Armini A.M., Morsolini M., Snijder R. (2011). Chronic Thromboembolic Pulmonary Hypertension (CTEPH). Circulation.

[13] Park S.Y., Lee S.M., Shin J.W., Choi B.W., Kim H., Lee J.S., Park S.S., Moon H.S., Park Y.B. (2016). Epidemiology of chronic thromboembolic pulmonary hypertension in Korea: Results from the Korean registry. Korean J. Intern. Med..

[14] Delcroix M., Lang I., Pepke-Zaba J., Jansa P., D’Armini A.M., Snijder R., Bresser P., Torbicki A., Mellemkjaer S., Lewczuk J. (2016). Long-Term Outcome of Patients With Chronic Thromboembolic Pulmonary Hypertension. Circulation.

[15] Delcroix M., Kerr K., Fedullo P. (2016). Chronic Thromboembolic Pulmonary Hypertension. Epidemiol. Risk Factors. Ann. Am. Thorac. Soc..

[16] Gerges C., Pistritto A.M., Gerges M., Friewald R., Hartig V., Hofbauer T.M., Reil B., Engel L., Dannenberg V., Kastl S.P. (2023). Left Ventricular Filling Pressure in Chronic Thromboembolic Pulmonary Hypertension. J. Am. Coll. Cardiol..

[17] Galiè N., Humbert M., Vachiery J.L., Gibbs S., Lang I., Torbicki A., Simonneau G., Peacock A., Noordegraaf A.V., Beghetti M. (2016). 2015 ESC/ERS Guidelines for the diagnosis and treatment of pulmonary hypertension. Eur. Heart J..

[18] Galiè N., Hoeper M.M., Humbert M., Torbicki A., Vachiery J.-L., Barbera J.A., Beghetti M., Task Force for Diagnosis and Treatment of Pulmonary Hypertension of European Society of Cardiology (ESC), European Respiratory Society (ERS), International Society of Heart and Lung Transplantation (ISHLT) (2009). Guidelines for the diagnosis and treatment of pulmonary hypertension. Eur. Respir. J..

[19] Charlson M.E., Pompei P., Ales K.L., MacKenzie C.R. (1987). A new method of classifying prognostic comorbidity in longitudinal studies: Development and validation. J. Chronic Dis..

[20] Elixhauser A., Steiner C., Harris D.R., Coffey R.M. (1998). Comorbidity Measures for Use with Administrative Data. Med. Care.

