# Supplementary material for: Comorbidity Burden in Chronic Thromboembolic Pulmonary Hypertension: Implications and Outcome
Source: Medicina (Kaunas). 2025 Apr 30;61(5):827. doi: 10.3390/medicina61050827 (PMC12113207; doi:10.3390/medicina61050827)
Supplement: Supplementary file 1 [file medicina-61-00827-s001.zip › medicina-3556164-supplementary.pdf]

Supplementary Table S1. Co-morbidities according to PEA status.

	Total N=187	PEA (+) N=64	PEA (-) N= 123	P
Comorbidities				
Sytemic hypertension	86 (46.0)	21 (32.8)	65 (53.3)	0.006
Diabetes mellitus	44 (23.5)	11 (17.5)	33 (26.8)	0.106
Smoking	39 (21.3)	14 (22.2)	25 (20.8)	0.827
Obesity	65 (34.8)	23 (38.3)	42 (39.3)	0.521
Coronary artery disease	30 (16.0)	3 (4.7)	27 (22.1)	0.001
Atrial fibrillation	27 (14.4)	6 (9.5)	21 (17.5)	0.108
Heart Failure	33 (17.6)	6 (9.4)	27 (22.1)	0.022
Stroke	10 (5.3)	5 (7.8)	5 (4.1)	0.234
Lung disease	69 (36.9)	15 (23.4)	54 (44.6)	0.003
COPD	52 (27.8)	8 (12.5)	44 (37.3)	< 0.001
Anemia	66 (35.3)	19 (31.1)	47 (39.5)	0.175
Chronic kidney disease	36 (19.3)	7 (10.9)	29 (23.6)	0.027
Connective tissue disease	12 (6.4)	11 (17.2)	1 (0.8)	< 0.001
Active cancer	5 (2.7)	1 (1.6)	4 (3.3)	0.441
Thyroid replacement therapy	13 (7.0)	6 (9.4)	7 (5.7)	0.257
Comorbidity Burden				
Number of cardiovascular comorbidities				
0	41 (22.2)	19 (29.7)	22 (18.2)	
1-2	87 (46.5)	32 (50.0)	55 (44.7)	0.033
≥3	59 (31.6)	13 (20.3)	46 (37.4)	
Number of non-cardiovascular comorbidities				
0	61 (32.6)	27 (42.2)	34 (27.6)	
1-2	116 (62.0)	37 (57.8)	79 (64.2)	0.004
≥3	10 (5.3)	0 (0)	10 (8.1)	
Any CV and non-CV comorbidity				
0	16 (8.6)	10 (15.6)	6 (4.9)	
1	36 (19.3)	12 (18.8)	24 (19.5)	
2	43 (23.0)	18 (28.1)	25 (20.3)	
3	37 (19.8)	15 (23.4)	22 (17.9)	
4	19 (10.2)	5 (7.8)	14 (11.4)	0.001
5	20 (10.7)	1 (1.6)	19 (15.4)	
6	6 (3.2)	1 (1.6)	5 (4.1)	
7	8 (4.3)	2 (3.1)	6 (4.9)	
8	2 (1.1)	0 (0)	2 (1.6)	
Total CV comorbidity	1.8 ± 1.5	1.3 ± 1.3	2.0 ± 1.5	0.001
Total non-CV comorbidity	1 ± 0.9	0.8 ± 0.8	1.1 ± 0.9	0.028

CV: cardiovascular, PEA: pulmonary endarterectomy

Supplementary Table S2. Initial six-minute walk distance by comorbidity status.

	Yes [N]	No [N].029	P	Cohen's d (95% CI)
**Hypertension**	247 ± 139 [77]	297 ± 153 [89]	0.029	0.343 (0.035 - 0.650)
**Diabetes mellitus**	220 ± 130 [41]	289 ± 150 [125]	0.009	0.473 (0.116 - 0.829)
**Obesity**	265 ± 135 [58]	300 ± 154 [91]	0.161	
**Smoking**	244 ± 159 [35]	281 ± 146 [131]	0.187	
**Coronary artery dis.**	207 ±138 [26]	285 ± 147 [141]	0.013	0.536 (0.113 - 0.958)
**Atrial Fibrillation**	233 ± 138 [25]	280 ± 150 [139]	0.148	
**Heart Failure**	244 ± 125 [33]	280 ± 153 [134]	0.213	
**Stroke**	199 ± 121 [9]	279 ± 149 [157]	0.119	.
**Pulmonary disease**	255 ± 146 [62]	283 ±150 [104]	0.235	
**Chronic kidney disease**	209 ± 132 [34]	289 ± 146 [133]	0.005	0.550 (0.168 - 0.930)
**Anemia**	260 ± 156 [58]	280 ± 146 [105]	0.411	
**Connective tissue dis.**	289 ± 156 [12]	272 ± 148 [155]	0.706	
**Active cancer**	208 ± 140 [5]	275 ± 149 [162]	0.320	.
**Thyroid replac. theraphy**	261 ± 116 [13]	274 ± 151 [154]	0.761	

Supplementary Table S3. Baseline hemodynamic parameters according to comorbidity status.

Comorbidity	Parameter	Yes	No	P	Cohen's d (95%CI)
**Hypertension**	RAP	11.7 ± 5.0	10.3 ± 4.5	0.038	-0.309 (-0.600 - -0.017)
	sPAP	65.6 ± 19.3	68.8 ± 21.4	0.287	
	mPAP	40.7 ± 11.2	42.1 ± 11.8	0.394	
	PAWP	12.7 ± 5.4	10.7 ± 4.1	0.006	-0.419 (-0.715 - -0.122)
	PVR	7.2 ± 4.8	8.2 ± 4.1	0.126	
	CI	2.5 ± 0.8	2.3 ± 0.7	0.326	
**Diabetes mellitus**	RAP	12.1 ± 4.6	10.5 ± 4.8	0.058	-0.332 (-0.675 - 0.011)
	sPAP	69.7 ± 21.0	66.5 ± 20.4	0.364	
	mPAP	43.3 ± 12.9	40.8 ± 10.4	0.232	
	PAWP	11.9 ± 5.3	11.6 ± 4.7	0.676	
	PVR	8.7 ± 6.0	7.5 ± 3.8	0.221	
	CI	2.2 ± 0.6	2.5 ±0.8	0.064	0.426 (0.053 - 0.795)
**Obesity**	RAP	11.0 ± 5.0	11.1 ± 4.8	0.827	
	sPAP	64.6 ± 19.6	67.6 ± 21.0	0.362	
	mPAP	39.8 ± 10.0	42.2 ± 11.8	0.096	
	PAWP	12.0 ± 5.3	12.0 ± 4.5	0.482	
	PVR	6.6 ± 0.8	8.2 ± 4.4	0.020	0.373 (0.056 - 0.688)
	CI	2.4 ± 0.8	2.4 ± 0.8	0.968	
**Smoking**	RAP	11.8 ± 5.4	10.6 ± 4.6	0.206	
	sPAP	69.6 ± 23.6	66.3 ± 19.6	0.425	
	mPAP	42.1 ± 11.9	41.1 ± 10.8	0.613	
	PAWP	11.5 ± 4.4	11.7 ± 4.9	0.861	
	PVR	7.3 ± 3.5	7.8 ± 4.7	0.574	
	CI	2.4 ± 0.8	2.4 ± 0.8	0.888	
**Coronary artery disease**	RAP	12.1 ± 4.2	10.7 ± 4.9	0.145	
	sPAP	65.6 ± 21.6	67.4 ± 20.4	0.697	
	mPAP	40 ± 10.6	41.6 ± 11.1	0.488	
	PAWP	11.8 ± 4.5	11.6 ± 4.9	0.793	
	PVR	8.1 ± 5.6	7.7 ± 4.2	0.589	
	CI	2.2 ± 0.7	2.4 ± 0.8	0.171	
**Atrial Fibrillation**	RAP	12.7 ± 4.8	10.6 ± 4.7	0.028	
	sPAP	66.1 ± 19.3	67.5 ± 21.0	0.744	
	mPAP	42.9 ± 10.3	41.1 ± 11.2	0.438	-0.461 (-0.872 - -0.049)
	PAWP	13.1 ± 5.7	11.2 ± 4.4	0.110	
	PVR	8.3 ± 5.3	7.7 ± 4.3	0.493	
	CI	2.4 ± 1.0	2.4 ± 0.7	0.916	
**Heart Failure**	RAP	14.2 ± 4.4	10.2 ± 4.6	< 0.001	-0.887 (-1.273 - -0.499)
	sPAP	66.7 ± 18.7	67.3 ± 20.9	0.884	
	mPAP	43.1 ± 11.6	41.0 ± 10.9	0.326	
	PAWP	18.3 ± 5.7	10.1 ± 3.0	< 0.001	-2.256 (-2.698 - -1.809)
	PVR	6.1 ± 2.9	8.1 ± 4.7	0.003	0.446 (0.066 - 0.824)
	CI	2.2 ± 0.6	2.4 ± 0.8	0.068	0.302 (-0.080 - 0.684)
**Stroke**	RAP	13.5 ± 6.2	10.8 ± 4.7	0.200	
	sPAP	76.2 ± 15.9	66.5 ± 20.6	0.147	
	mPAP	45.8 ± 8.7	41.1 ± 11.1	0.189	
	PAWP	13.7 ± 6.4	11.5 ± 4.7	0.314	
	PVR	10.0 ± 6.7	7.6 ± 4.3	0.283	
	CI	2.1 ± 0.9	2.4 ± 0.8	0.253	
**Pulmonary disease**	RAP	10.9 ± 4.7	10.9 ± 4.9	0.988	
	sPAP	64.5 ± 20.9	68.7 ± 20.3	0.179	
	mPAP	39.8 ± 10.5	42.2 ± 11.3	0.159	
	PAWP	11.0 ± 4.3	11.9 ± 4.9	0.233	
	PVR	7.4 ± 4.1	8.0 ± 4.7	0.396	

	CI	2.4 ± 0.6	2.4 ± 0.9	0.597	
**Chronic kidney disease**	RAP	12.7 ± 4.8	10.5 ± 4.7	0.011	
	sPAP	70 ± 17.3	66.5 ± 21.2	0.383	-0.480 (-0.847 - -0.112)
	mPAP	43.4 ± 10.2	40.9 ± 11.2	0.211	
	PAWP	13.4 ± 6.1	11.2 ± 4.4	0.045	
	PVR	8.7 ± 4.6	7.5 ± 4.4	0.139	-0.473 (-0.845 - -0.100)
	CI	2.6 ± 0.8	2.3 ± 0.8	0.120	
**Anemia**	RAP	11.4 ± 4.8	10.5 ± 4.7	0.221	
	sPAP	63.9 ± 18.8	69.0 ± 21.5	0,115	
	mPAP	39.6 ± 10.2	42.1 ± 11.5	0,153	
	PAWP	12.5 ± 5.4	10.9 ± 4.1	0.041	-0.346 (-0.655 - -0.036)
	PVR	6.9 ± 43.	8.1 ± 4.2	0.070	0.284 (-0.023 - 0.590)
	CI	2.4 ± 0.7	2.4 ± 0.8	0.845	
**Connective tissue disease**	RAP	12.2 ± 6.2	10.8 ± 4.7	0.473	
	sPAP	69.9 ± 22.8	67.0 ± 20.4	0.633	
	mPAP	43.1 ± 12.5	41.2 ± 10.9	0.574	
	PAWP	10.2 ± 4.1	11.7 ± 4.9	0.279	
	PVR	8.4 ± 4.8	7.7 ± 4.4	0.613	
	CI	2.2 ± 0.6	2.4 ± 0.8	0.348	
**Active cancer**	RAP	12.6 ± 6.7	10.9 ± 4.7	0.422	
	sPAP	72.2 ± 19.4	67 ± 20.6	0.580	
	mPAP	40 ± 10.8	41.4 ± 11.1	0.782	
	PAWP	14.6 ± 4.8	11.5 ± 4.8	0.161	
	PVR	5.0 ± 1.6	7.8 ± 4.5	0.011	0.632 (-0.260 – 1.522)
	CI	2.0 ± 0.7	2.4 ± 0.8	0.237	
**Thyroid replacement therapy**	RAP	10.4 ± 5.1	10.9 ± 4.8	0.690	
	sPAP	72.8 ± 21.5	66.8 ± 20.4	0.309	
	mPAP	45.4 ± 14.3	41.1 ± 10.7	0.171	
	PAWP	11.6 ± 4.5	11.6 ± 4.8	0.977	
	PVR	8.4 ± 3.7	7.7 ± 4.5	0.582	
	CI	2.6 ± 0.8	2.4 ± 0.8	0.433	

Supplementary Table S4. Characteristics of survivors and non-survivors in PEA (+) group.

	N/N	Survivors N = 54	Nonsurvivors N = 7	p
**Age**	54/7	49.7 ± 13.8	61.4 ± 12.7	0.037
**Female**	54/7	32 (59.3)	5 (71.4)	0.535
**Previous acute PE history**	52/6	48 (92.3)	6 (100)	1.000
**WHO-FC I – II**	54/7	13 (24.1)	0 (0)	0.328
**III – IV**		41 (75.9)	7 (100)	
**Initial 6MWD, m [51/6]]**	51/6	325.3 ± 143.5	178.8 ± 77.8	0.003
**First year 6MWD, m [43/7]**	43/7	425.7 ± 111.4	206.6 ± 176.9	< 0.001
**Δ6MVD, m**	42/6	96.2 ± 119.4	62.2 ± 212.5	0.560
**Mean RA pressure, mmHg**	54/7	11.1 ± 5.4	11.0 ± 4.2	0.979
**PA systolic pressure, mmHg**	54/7	75.8 ± 20.6	83.3 ± 24.8	0.379
**PA mean pressure, mmHg**	54/7	45.7 ± 11.5	52.0 ± 13.2	0.188
**PA wedge pressure, mmHg**	52/6	11.0 ± 4.0	12 ± 4.0	0.571
**PVR, Wood units**	54/6	9.1 ± 4.6	8.6 ± 2.2	0.771
**Cardiac index, L/min/m^2**	53/7	2.3 ± 0.7	2.0 ± 0.4	0.254
**Pulmonary specific medication**	54/7	30 (55.6)	6 (85.7)	0.223
**Warfarin**	54/7	32 (59.3)	2 (28.6)	0.224
**DOAC**	54/7	15 (27.8)	6 (85.7)	0.005
**Furosemid**	54/7	15 (27.8)	3 (42.9)	0.411
**Spironolactone**	54/7	8 (14.8)	2 (28.6)	0.322
**Sytemic hypertension**	54/7	15 (27.8)	3 (42.9)	0.411
**Diabetes mellitus**	53/7	7 (13.2)	3 (42.9)	0.083
**Smoking**	53/7	11 (20.8)	3 (42.9)	0.337
**Obesity**	50/7	19 (38.0)	4 (57.1)	0.423
**Coronary artery disease**	54/7	1 (1.9)	2 (28.6)	0.032
**Atrial fibrillation**	53/7	3 (5.7)	3 (42.9)	0.017
**Heart Failure**	54/7	4 (7.4)	1 (14.3)	0.468
**Stroke**	54/7	4 (7.4)	0 (0)	1.000
**Lung disease**	54/7	10 (18.5)	3 (42.9)	0.159
**Anemia**	51/7	17 (33.3)	2 (28.6)	1.000
**Chronic kidney disease**	54/7	4 (7.4)	2 (28.6)	0.136
**Connective tissue disease**	54/7	11 (20.4)	0 (0)	0.332
**Active cancer**	54/7	1 (1.9)	0 (0)	1.000
**Thyroid replacement therapy**	54/7	5 (9.3)	1 (14.3)	0.535
**Total number of comorbidities**	54/7	1.9 ± 1.4	3.7 ± 2.3	0.002
**≥3 comorbidites**	54/7	16 (29.6)	6 (85.7)	0.007
**≥3 CV comorbidities**	54/7	8 (14.8)	4 (57.1)	0.023
**≥3 non-CV comorbidities**	54/7	0	0	-

Supplementary Table S5. Characteristics of survivors and non-survivors in PEA (-) group.

	N/N	Survivors 89	Nonsurvivors 34	p
**Age**	89/34	63.9 ± 12.9	66.9 ± 12.4	0.247
**Female**	89/34	59 (66.3)	21 (61.8)	0.638
**Previous acute PE history**	82/31	48 (58.5)	23 (74.2)	0.124
**WHO-FC I -II**	89/34	18 (20.2)	4 (11.8)	0.430
**III – IV**		71 (79.8)	30 (88.2)	
**Initial 6MWD, m [82/26]**	82/26	269.0 ± 152.3	216.5 ± 123.3	0.056
**First year 6MWD, m [65/19]**	65/19	315.3 ± 124.2	246.1 ± 143.4	0.021
**Δ6MVD, m [62/16]**	63/16	51.2 ± 103.1	16.2 ± 159.8	0.286
**Mean RA pressure, mmHg**	87/34	11.0 ± 4.6	10.1 ± 4.4	0.150
**PA systolic pressure, mmHg**	89/34	59.2 ± 18.1	69.8 ± 18.1	0.004
**PA mean pressure, mmHg**	89/34	37.3 ± 9.5	42 ± 8.9	0.014
**PA wedge pressure, mmHg**	86/33	11.6 ± 4.8	12.5 ± 5.9	0.401
**PVR, Wood units**	88/33	6.5 ± 3.9	8.4 ± 5.2	0.041
**Cardiac index, L/min/m^2**	78/26	2.5 ± 0.8	2.4 ± 0.9	0.953
**Pulmonary specific medication**	89/34	74 (83.1)	27 (79.4)	0.629
**Warfarin**	88/32	19 (21.6)	7(21.9)	0.973
**DOAC**	88/33	62 (70.5)	24 (72.7)	1.000
**Furosemid**	87/32	28 (32.2)	14 (43.8)	0.242
**Spironolactone**	87/32	31 (35.6)	8 (25.0)	0.273
**Sytemic hypertension**	88/34	51 (58.0)	14 (41.2)	0.096
**Diabetes mellitus**	89/34	26 (29.2)	7 (20.6)	0.334
**Smoking**	89/34	18 (20.2)	7 (20.6)	0.964
**Obesity**	82/25	34 (41.5)	8 (32.0)	0.396
**Coronary artery disease**	89/34	23 (25.8)	4 (11.8)	0.142
**Atrial fibrillation**	88/32	19 (21.6)	2 (6.3)	0.059
**Heart Failure**	89/34	20 (22.5)	7 (20.6)	0.821
**Stroke**	88/34	4 (4.5)	1 (2.9)	1.000
**Lung disease**	89/33	46 (51.7)	8 (24.2)	0.007
**Anemia**	88/32	33 (37.5)	14 (43.8)	0.535
**Chronic kidney disease**	89/34	20 (22.5)	9 (26.5)	0.640
**Connective tissue disease**	89/34	1 (1.1)	0 (0)	1.000
**Active cancer**	89/34	3 (3.4)	1 (2.9)	1.000
**Thyroid replacement therapy**	89/34	6 (6.7)	1 (2.9)	0.672
**Total number of comorbidities**	89/34	3.3 ± 2.0	2.4 ± 1.5	0.007
**≥3 comorbidites**	89/34	55 (61.8)	13 (38.2)	0.019
**≥3 CV comorbidities**	87/34	37 (42.5)	8 (23.5)	0.052
**≥3 non-CV comorbidities**	89/34	9 (10.1)	1 (2.9)	0.281
